# Vitamin D and Sun Exposure: A Community Survey in Australia

**DOI:** 10.3390/curroncol30020188

**Published:** 2023-02-18

**Authors:** Vu Tran, Monika Janda, Robyn M. Lucas, Donald S. A. McLeod, Bridie S. Thompson, Mary Waterhouse, David C. Whiteman, Rachel E. Neale

**Affiliations:** 1 QIMR Berghofer Medical Research Institute, Brisbane, QLD 4006, Australia; 2 The University of Queensland, Brisbane, QLD 4006, Australia; 3 The Australian National University, Canberra, ACT 2601, Australia; 4 Royal Brisbane and Women’s Hospital, Brisbane, QLD 4029, Australia

**Keywords:** skin cancers, vitamin D, sun exposure, sun protection, knowledge, attitudes

## Abstract

Sun exposure carries both harms and benefits. Exposing the skin to the sun is the main modifiable cause of skin cancers, which exert a considerable health and economic burden in Australia. The most well-established benefit of exposure to ultraviolet (UV) radiation is vitamin D production. Australia has the highest incidence of skin cancer in the world but, despite the high ambient UV radiation, approximately one quarter of the population is estimated to be vitamin D deficient. Balancing the risks and benefits is challenging and requires effective communication. We sought to provide a snapshot of public knowledge and attitudes regarding sun exposure and vitamin D and to examine the associations between these factors and sun protective behaviors. In 2020 we administered an online survey; 4824 participants with self-reported fair or medium skin color were included in this analysis. Only 25% and 34% of participants were able to identify the amount of time outdoors needed to maintain adequate vitamin D status in summer and winter, respectively and 25% were concerned that sunscreen use inhibits vitamin D synthesis. This lack of knowledge was associated with suboptimal sun protection practices. Public education is warranted to prevent over-exposure, while supporting natural vitamin D production.

## 1. Introduction

Skin cancers impose a considerable health and economic burden in many countries, particularly those with high ambient ultraviolet (UV) radiation and a high proportion of fair-skinned people, such as Australia and New Zealand [1,2]. The majority of melanomas and keratinocyte cancers are attributable to exposure to solar UV radiation [3]. Thus, protecting the skin through avoiding time outdoors when the UV index is high, wearing hats and protective clothing and applying sunscreen are a priority for skin cancer prevention [3,4]. In Australia, formal sun protection campaigns began with the SunSmart programs in the 1980s. These have led to reduced frequency of sunburns [5] and, in more recent birth cohorts who were exposed to the campaigns from childhood, skin cancer rates are dropping [6,7].

Although exposure to UV radiation can cause considerable harm, it also offers health benefits, of which the most well-established is cutaneous vitamin D synthesis. Vitamin D plays an important role in musculoskeletal health and there is evidence that low vitamin D status may increase the risk of other health outcomes such as infection, autoimmune disease and mortality [8,9,10]. Exposure to UV radiation also has other benefits through non-vitamin D pathways, such as UVA-induced release of nitric oxide into the circulation which lowers blood pressure and may reduce the risk of metabolic disorders [11,12,13] and systemic immunosuppression that may reduce the risk of autoimmune diseases [13,14]. In addition, exposure to non-UV wavelengths in sunlight reduces the risk and possibly progression of myopia and influences circadian rhythm, sleep and mood [15,16]. Thus, avoiding sun exposure might have undesirable consequences, even in the presence of vitamin D supplementation.

It is challenging to balance the benefits and harms of sun exposure. Despite Australia’s high ambient UV radiation, approximately a quarter of the population was estimated to be vitamin D deficient (serum 25-hydroxyvitamin D concentration <50 nmol/L) in the 2011 National Health Survey [17]. There is consensus that frequent sub-erythemal doses of UV radiation with ample skin exposed is optimal for maintaining adequate vitamin D status [18,19] and current evidence suggests that frequent use of sunscreen does not lead to vitamin D deficiency [20]. However, guidelines about balancing the risks and benefits of sun exposure vary [21] and there is evidence that primary care practitioners and the general public lack clarity on this issue [22,23].

The balance of the risks and benefits of sun exposure varies according to skin type. People with highly pigmented skin (Fitzpatrick skin types V and VI) have much lower risk of both melanoma [24] and keratinocyte cancer [25] due to the high protection against UV-induced DNA damage afforded by melanin [26]. In contrast, multiple studies, including in Australia, demonstrate that the prevalence of vitamin D deficiency in people with highly pigmented skin is higher than in paler-skinned people living in the same location [27,28,29]. The additional dose of UV radiation (and therefore time outdoors) needed to maintain adequate vitamin D in darker-skinned individuals is not certain, but ranges from a 1.3-fold increase comparing skin type VI with skin type II in one study [30] to a 7-fold increase comparing skin type VI with skin type I in another [31]. In light of these differing risks, people with darker skin types can spend time outdoors to maintain adequate vitamin D status with negligible increased risk of skin cancer. Irrespective of skin type, health conditions, occupation, clothing or lifestyle choices may result in some people being unable to maintain adequate vitamin D status through sun exposure. In this case, vitamin D requirements can be met through supplementation, with guidelines in adults ranging from 400 IU [32] to 800 IU per day [33].

Multiple surveys have investigated public knowledge regarding vitamin D and sun protection [34,35,36,37,38,39,40,41,42,43]. Most found that a substantial proportion of respondents believed that regular sun protection causes vitamin D deficiency [35,36,42,43,44] and one study identified that this perception was the most important factor influencing suboptimal sun protection behaviors [41]. Only two surveys considered associations between knowledge of the amount of time outdoors required to avoid vitamin D deficiency and sun exposure behaviors [36,37].

We used data from an online survey carried out in Australia in the summer (January) of 2020 to: (1) obtain a recent indication of public knowledge about/attitudes towards vitamin D and sun exposure and their associated factors; (2) examine the association between knowledge/attitudes and sun-related behaviors; and (3) examine the association between knowledge/attitudes and being sunburnt.

## 2. Materials and Methods

### 2.1. Design and Procedure

We used data from the *Vitamin D and sun exposure: community survey* which was carried out by the QIMR Berghofer Medical Research Institute (QIMR Berghofer) in Australia in January 2020. Participants were aged 18 years or more and recruited via social media and through emailed invitations to people who had previously completed the QIMR Berghofer online melanoma risk prediction tool [45,46] and had given consent to be contacted about research studies. The study was approved by the QIMR Berghofer Human Research Ethics Committee. Participants were provided with a plain language statement and, by completing the online survey, gave implied consent.

The survey asked about personal characteristics including: age group, sex, Australian state or territory of residence, skin color, propensity to sunburn, educational attainment, occupational status and history of skin cancer diagnosis and treatment. Due to smaller numbers in some states/territories we grouped these into 4 categories based on their close proximity and similar latitude (noting that 70% of residents of Western Australia live in the south of the state); i.e., Queensland/Northern Territory, Australian Capital Territory/New South Wales, Victoria/Tasmania and Western Australian/South Australia.

We assessed participants’ knowledge of the time needed outdoors to maintain adequate vitamin D status by asking “Imagine that you are wearing shorts and a short-sleeved shirt. How much time do you think you need to spend outdoors (where you live) between 9am and 3pm each day to avoid having low vitamin D in: (i) summer; and (ii) winter?”. We used estimates of the actual time needed to maintain adequate vitamin D from the Position Statement for Vitamin D and Health in Adults in Australia and New Zealand [47], allowing for some uncertainty, to classify answers into correct, over-estimate (for summer and winter) and under-estimate for winter (the thresholds used are shown in Table 1).

Concern about the effect of sunscreen use on vitamin D production was assessed by asking respondents about their agreement, on a 5-point Likert scale, with the statement “If I wear sunscreen when I am outdoors it will stop my skin from making vitamin D”. With regards to sun exposure behaviors, we asked participants whether they had changed their behavior to obtain more vitamin D and, if so, what had changed (spending more time outdoors, wearing a hat less often, applying sunscreen less, sunbathing more often and using a sunbed). Routine sunscreen use was assessed by asking “Do you routinely apply (that is, on most days) sunscreen, including moisturizers or makeup with a sun protection factor, regardless of whether or not you are going out in the sun?”, with answers specific to face, hands/forearms, or other parts of the body. Separately, we also asked participants how frequently they applied sunscreen, wore a hat and wore long sleeves when they planned to be outdoors for 30 min or more in summer. For participants who reported never/rarely wearing sunscreen, we asked for reasons, with multiple selections allowed.

Frequency of sunburn, which was defined on the survey as “reddening of the skin that lasts more than 12 h after exposure to the sun”, in the last 6 months was categorized into: never, 1–2 times, 3–4 times, 5–9 times and 10 or more times. As there were few participants who reported having being burnt more than twice we recoded this as no burns versus one or more burns.

### 2.2. Statistical Analysis

We described the differences between skin type groups with regards to personal characteristics, knowledge, attitudes and behaviors (Supplementary Table S1). However, as the balance of the risks and benefits of sun exposure differs according to skin type, we restricted further analyses to people who reported having fair or medium skin who are at markedly higher risk for skin cancer. Analyses of those with olive/dark skin will be presented elsewhere. A priori we considered state/territory of residence, age, sex, skin color and educational attainment to be covariates in multivariable analysis, so participants with missing data for any of these variables were excluded from the analysis.

We described participant characteristics and knowledge, attitudes and behavior variables using numbers and simple percentages. To estimate associations between: (1) personal characteristics and knowledge/attitudes; (2) personal characteristics and behavior; (3) knowledge/attitudes and behavior; and (4) knowledge/attitudes and being sunburnt, we calculated prevalence ratios (PRs) and 95% confidence intervals (95% CI) using log-binomial regression. In cases where the log-binomial model did not converge or was unable to estimate the covariance matrix, we used Poisson regression.

We used directed acyclic graphs to help identify additional potential confounders of the associations of interest, including propensity to sunburn, occupational status and history of skin cancer treatment. In multivariable analysis, we first applied a change in estimate approach (>10%) to decide whether to include or exclude a covariate in the final model. In addition, we repeated analyses separately for men and women to examine whether the associations of interest differed by sex. All analyses were performed using R version 4.1.2.

## 3. Results

### 3.1. Characteristics of Study Sample

Of the 5602 participants who completed the survey, we excluded three participants who reported having black skin and 228 (4.1%) who did not report their skin type. The distribution of demographic, phenotypic, knowledge, attitudes and behaviors according to skin type is shown in Supplementary Table S1. The percentage of people who reported a past history of skin cancer, for instance melanoma, was lower in those with olive/dark skin (5.6%) than in those with medium (11.9%) or fair (16.7%) skin. Participants with olive/dark skin were less likely to report using sun protection strategies than those with fair or medium skin. 

**Figure 1:**
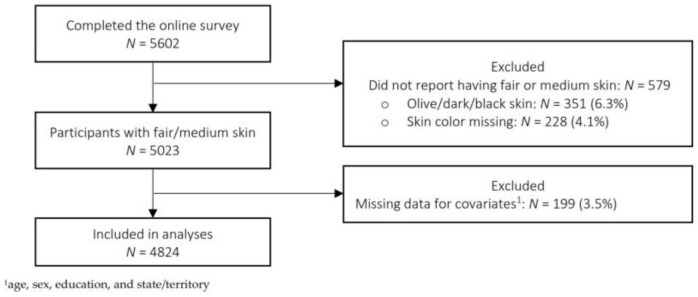
Flowchart of participant selection for analysis.

After excluding those with olive/dark skin (*N* = 348) and who were missing core covariates (*N* = 199) 4824 participants remained in the analysis (Figure 1). There were no significant differences in terms of age, sex, state/territory of residence, history of skin cancer, vitamin D supplement use and testing between those who were included and excluded (data not presented). Similarly, the results of analyses stratified by sex were not different compared to the main analyses (data not presented).

Participant characteristics are described in Table 2. Just over half the participants were aged 60 years or older, 68% were women and 60% had a university degree. Nearly three quarters described their natural skin color on areas rarely exposed to the sun as “fair” and 58% had skin that would be moderately or badly burnt if exposed to the sun for 30 min without any protection. Forty-two percent reported a history of skin cancer excision and 55% reported having skin lesions treated by cryotherapy. These percentages were higher in Queensland and the Northern Territory (lower latitude) compared with other states/territory. The percentage of participants who were taking vitamin D supplements was highest in the southern states (higher latitude) of Victoria and Tasmania (37%) and lowest in Queensland and the Northern Territory (26%).

### 3.2. Knowledge, Attitudes towards Vitamin D and Sun Exposure

Overall, 76% of respondents over-estimated the time outdoors needed to maintain adequate vitamin D in summer; no participants under-estimated the time required (Supplementary Table S2). For winter, 37% of people over-estimated and 32% under-estimated the time required (Supplementary Table S2). Participants from Victoria or Tasmania were more likely to over-estimate the time needed in summer, while participants from other states/territory were less likely than those from Queensland or the Northern Territory to over-estimate in winter.

Approximately a quarter of respondents believed that sunscreen use prevents cutaneous vitamin D production (Supplementary Table S2). Participants who were not from Queensland or the Northern Territory and those who were older, female, had fair skin, higher educational attainment and no history of skin cancer excision were more likely to agree with this statement compared with those who did not have these characteristics.

The results of multivariable analyses of the associations between personal characteristics and knowledge about time outdoors needed to avoid vitamin D deficiency are shown in Table 3. We found that men, those with less sun-sensitive phenotypes and people with lower educational attainment were more likely to over-estimate the time required in both seasons, compared with those in alternative categories of these variables. Participants from Victoria or Tasmania (versus Queensland/Northern Territory), who were older, or who had no history of treatment for melanoma were more likely to over-estimate the time in summer. Participants from Queensland or the Northern Territory (compared with those in other states/territories), or younger (<40 versus 40–59 years) were more likely to over-estimate the needed time in winter. The risk of under-estimating the time required in winter was lower among men, older participants, those from Victoria/Tasmania (versus Queensland/Northern Territory), those with less sun-sensitive skin types and people who were less educated or had never been treated for melanoma compared with participants from other categories.

### 3.3. Sun-Related Behaviors

**Figure 2:**
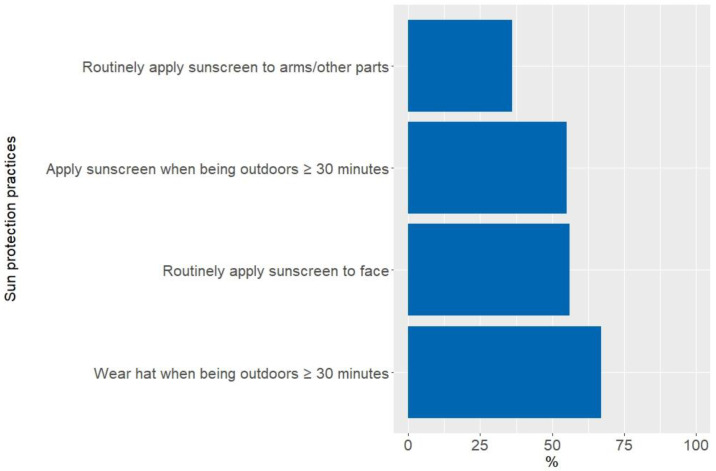
Proportion of sun protection practices reported by the participants.

In total, 67% and 55% of respondents reported wearing a hat and applying sunscreen almost all or all of the time when outdoors for ≥30 min in summer, respectively (Figure 2). Routine sunscreen application to the face was reported by 56% of participants and 36% reported routine application to other body parts. Women and those with sun-sensitive phenotypes were more likely to practice sun protection measures than men and those with less sensitive skin (Supplementary Table S3). An exception to this was that men were more likely to wear a hat than women (Supplementary Table S3).

Over-estimating the time outdoors needed to maintain vitamin D in summer was negatively associated with wearing a hat (adjusted PR, APR = 0.86, 95% CI = 0.82–0.89) and applying sunscreen when outdoors for ≥30 min (APR = 0.85, 95% CI = 0.81–0.89). Similarly, participants who over-estimated the time required in winter were less likely to apply sunscreen when outdoors for ≥30 min (APR = 0.81, 95% CI = 0.76–0.87) (Table 4).

Overall, 14% of respondents (*N* = 686) reported that they had changed their sun exposure behavior in order to maintain adequate vitamin D status. Of those, 72% spent more time outdoors, 17% applied sunscreen less and 6% sunbathed more often. Women were more likely to report changing their behaviors than men (16% vs. 10%, Supplementary Table S4). Among those who changed their behaviors, participants aged less than 40 years were more likely than those aged 40 years or older to report spending more time outdoors and less likely to report reducing their sunscreen use (Supplementary Table S4).

Table 5 displays the results of the multivariable analysis of associations between knowledge about/attitudes towards vitamin D, vitamin D testing and supplement use and changing sun exposure behaviors. Participants who were taking a vitamin D supplement, who had their vitamin D tested in the past 12 months, or who believed that sunscreen stops cutaneous vitamin D synthesis were more likely to report having changed their sun exposure behaviors than those who were not in these categories. Of note, agreeing that sunscreen prevents vitamin D production was associated with applying sunscreen less (APR = 3.20, 95% CI = 2.25–4.55) and sunbathing more often (APR = 1.66, 95% CI = 0.93–2.97), while those taking a vitamin D supplement were less likely to report applying sunscreen less (APR = 0.63, 95% CI = 0.43–0.92).

### 3.4. Sunburn

In total, 37% of participants reported being sunburnt in the past 6 months. This percentage was higher among men, those in younger age groups, who were currently working, had skin that was more susceptible to sunburn and who did not have a history of skin cancer treatment (Supplementary Table S4).

Over-estimating the time needed to maintain adequate vitamin D in summer (APR = 1.27, 95% CI = 1.16–1.39) and winter (APR = 1.22, 95% CI = 1.12–1.33) was associated with greater risk of being sunburnt. People who had been tested for vitamin D (APR = 0.87, 95% CI = 0.80–0.95) or who were taking a vitamin D supplement (APR = 0.81, 95% CI = 0.74–0.88) were less likely to have been sunburnt in the previous six months (Table 6).

## 4. Discussion

In this survey of almost 5000 Australian adults, we observed that almost three-quarters of participants over-estimated the time needed in the sun to maintain adequate vitamin D status in summer and that 25% were concerned about the effect of sunscreen on vitamin D production. Importantly, this concern was adversely associated with sun-related practices and sunburn, which might place people at increased risk of skin cancer.

Finding the optimal amount of sun exposure for overall health is challenging and depends on a wide range of personal and environmental factors [48,49,50]. Guidelines from the Cancer Council Australia for people with fair or medium skin types recommend that in summer a few minutes outdoors mid-morning or mid-afternoon on most days of the week is sufficient to maintain adequate vitamin D throughout the whole of Australia [51]. In southern states such as Victoria, South Australia and Tasmania in winter, 2–3 h per week in the middle of the day is recommended. We found that three quarters of people over-estimated the time they needed to spend outdoors in summer and 37% over-estimated in winter. These figures are somewhat higher than those identified in an online survey of Queensland-based employees of a large banking and insurance organization, in which 56% and 28% over-estimated the time needed in summer and winter, respectively [37]. Of note, people from Victoria or Tasmania were slightly more likely to over-estimate the time needed in summer, whereas they were more likely to under-estimate the time needed in winter compared with those from the Northern Territory or Queensland; this potentially contributes to the high prevalence of vitamin D deficiency (concentration of serum 25(OH)D < 50 nmol/L) in winter, which approaches 50% in these states [52]. Those with a less sun-sensitive phenotype were more likely to over-estimate the time in both summer and winter, despite there being little evidence to support a difference in the time required to maintain vitamin D across Fitzpatrick skin types II to IV [29,30].

The lack of accurate assessment of the time needed to maintain adequate vitamin D status was associated with suboptimal sun protection behaviors. Those who over-estimated the time needed were more likely to report having changed their behavior to spend more time outdoors and were less likely to wear hats and sunscreen, supporting findings from previous surveys [36,37]. This link between knowledge and behavior clearly demonstrates the importance of education. However, a review of current guidelines from Australia and New Zealand found considerable variability in advice regarding the time required to maintain vitamin D [21], so the lack of knowledge within the Australian adult population surveyed here is unsurprising. Creating clear and consistent guidelines and implementing strategies to educate the public about this issue might reduce both over-exposure to the sun and vitamin D deficiency.

The Cancer Council Australia guidelines [51] state that regular use of sunscreen does not cause vitamin D deficiency. However, we found that approximately one-quarter of participants believed that applying sunscreen may lead to vitamin D deficiency, in line with a 2017 survey which found that 29% of study participants were concerned about the impact of sunscreen use on vitamin D [43]. Importantly, among participants who changed their exposure behaviors to ensure adequate vitamin D levels, those who were concerned about sunscreen and vitamin D were three times more likely to report having reduced their sunscreen use. Similarly, previous surveys found inverse associations between vitamin D concern and current sunscreen use [35,36,37,43]. The current guidelines are based on two field trials that found no difference in the 25(OH)D concentration between those randomized to daily sunscreen use and those randomized to control/placebo [53,54], even though sufficient sunscreen was applied to reduce the risks of skin cancer and/or premalignant lesions [55,56]. However, these trials used sunscreens with a sun protection factor (SPF) of approximately 16, which is considerably lower than the sunscreens now recommended and widely used. Given the importance of sunscreen use in reducing the incidence of skin cancer [3,57], it is important to clarify the effect of frequent use of high SPF sunscreen on vitamin D and then to derive policy and communication strategies that effectively address this issue.

Despite respondents being mostly well-educated and with an over-representation of people with a history of skin cancer, over a third reported a history of sunburn in the previous 6 months. Over-estimating the time outdoors needed to maintain adequate vitamin D status in both seasons was associated with an increased risk of sunburn, suggesting that education about the very short time outdoors needed, particularly in summer, may contribute to reduced risk of sunburn.

The association between attitudes towards vitamin D and sun-related practices, when taking into account vitamin D supplementation, is complex. We found that people who used vitamin D supplements were more likely to report changing their behavior to maintain adequate vitamin D status, staying outdoors more often, but being less likely to reduce their sunscreen use. It is plausible that vitamin D supplementation is an indicator of being concerned about vitamin D status, resulting in increased time outdoors and that it is used to compensate for a high frequency of sunscreen use. The cross-sectional nature of this study makes it difficult to disentangle the direction of these associations.

A key limitation of this study is the use of a volunteer sample, so attitudes and knowledge may not represent those in the overall Australian adult population. However, as this sample is highly educated, it likely under-estimates the lack of knowledge, highlighting the need for action in this area. Another limitation is reliance on self-report of factors such as phenotype, skin cancer history and sun-exposure behavior.

Skin cancer remains a major public health concern in Australia and sustained investment in sun protection campaigns is needed. Conversely, a significant proportion of people in southern states are vitamin D deficient in winter. These findings suggest that there is a need to incorporate education about the time outdoors need to maintain vitamin D status in both summer and winter and the safety of following advice to apply sunscreen routinely on all days when the UV index is forecast to reach 3 or above.

## Figures and Tables

**Table 1:** Definition of knowledge of time outdoors ^1^ (in minutes) needed to maintain adequate vitamin D in summer and winter.

State/Territory	Summer		Winter		
	Correct	Over-Estimate	Under-Estimate	Correct	Over-Estimate
Northern Territory	≤10	>10	≤10	>10 & ≤20	>20
Queensland	≤10	>10	≤10	>10 & ≤20	>20
New South Wales	≤10	>10	≤15	>15 & ≤30	>30
ACT	≤10	>10	≤15	>15 & ≤30	>30
Victoria	≤10	>10	≤15	>15 & ≤30	>30
Tasmania	≤10	>10	≤15	>15 & ≤30	>30
South Australia	≤10	>10	≤15	>15 & ≤30	>30
Western Australia	≤10	>10	≤15	>15 & ≤30	>30

^1^ Wearing shorts and short-sleeved shirt.

**Table 2:** Participant characteristics by state/territory of residence.

Personal Characteristics	*n* (Column %)				
	NT/QLD (*N* = 2219)	ACT/NSW (*N* = 1174)	VIC/TAS (*N* = 884)	WA/SA (*N* = 547)	Total (*N* = 4824)
Age group					
18–39	206 (9.3)	113 (9.6)	72 (8.1)	47 (8.6)	438 (9.1)
40–59	833 (37.5)	404 (34.4)	320 (36.2)	216 (39.5)	1773 (36.8)
60+	1180 (53.2)	657 (56.0)	492 (55.7)	284 (51.9)	2613 (54.2)
Sex					
Male	732 (33.0)	374 (31.9)	255 (28.8)	184 (33.6)	1545 (32.0)
Female	1487 (67.0)	800 (68.1)	629 (71.2)	363 (66.4)	3279 (68.0)
Skin color					
Fair	1571 (70.8)	797 (67.9)	630 (71.3)	378 (69.1)	3376 (70.0)
Medium	648 (29.2)	377 (32.1)	254 (28.7)	169 (30.9)	1448 (30.0)
Skin burning					
Burn badly	349 (15.7)	154 (13.1)	146 (16.5)	93 (17.0)	742 (15.4)
Burn moderately	945 (42.6)	509 (43.4)	373 (42.2)	232 (42.4)	2059 (42.7)
Burn a little	793 (35.7)	451 (38.4)	301 (34.1)	186 (34.0)	1731 (35.9)
Does not burn	132 (5.9)	60 (5.1)	63 (7.1)	36 (6.6)	291 (6.0)
Missing	0	0	1	0	1
Educational attainment					
No post-school qualification	443 (20.0)	144 (12.3)	117 (13.2)	99 (18.1)	803 (16.6)
Trade/apprenticeship/diploma	568 (25.6)	247 (21.0)	179 (20.2)	132 (24.1)	1126 (23.3)
University degree	1208 (54.4)	783 (66.7)	588 (66.5)	316 (57.8)	2895 (60.0)
Occupation status					
Full-time worker	752 (34.0)	343 (29.3)	246 (27.8)	159 (29.1)	1500 (31.1)
Part-time worker	385 (17.4)	201 (17.2)	195 (22.1)	106 (19.4)	887 (18.4)
Retired	913 (41.2)	516 (44.0)	357 (40.4)	222 (40.7)	2008 (41.7)
Other ^1^	165 (7.4)	112 (9.6)	86 (9.7)	59 (10.8)	422 (8.8)
Missing	4	2	0	1	7
Ever had melanoma removed ^2^					
No	1781 (81.6)	1004 (87.8)	782 (89.8)	449 (83.5)	4016 (84.8)
Yes	401 (18.4)	140 (12.2)	89 (10.2)	89 (16.5)	719 (15.2)
Missing	37	30	13	9	89
Number of skin cancers excised ^2^					
None	1089 (49.1)	686 (58.5)	601 (68.2)	333 (61.0)	2709 (56.2)
1	302 (13.6)	155 (13.2)	105 (11.9)	66 (12.1)	628 (13.0)
2+	827 (37.3)	332 (28.3)	175 (19.9)	147 (26.9)	1481 (30.7)
Missing	1	1	3	1	6
Number of skin cancers frozen/burnt ^2^					
None	885 (39.9)	516 (44.0)	515 (58.4)	264 (48.4)	2180 (45.3)
1–5	611 (27.6)	309 (26.3)	220 (24.9)	155 (28.4)	1295 (26.9)
6+	720 (32.5)	348 (29.7)	147 (16.7)	127 (23.3)	1342 (27.9)
Missing	3	1	2	1	7
Taking vitamin D supplement					
No	1615 (74.2)	797 (69.1)	550 (62.9)	365 (68.6)	3327 (70.2)
Yes	561 (25.8)	357 (30.9)	324 (37.1)	167 (31.4)	1409 (29.8)
Missing	43	20	10	15	88
Had vitamin D tested <12 months ago					
No	1351 (61.0)	603 (51.5)	497 (56.3)	310 (56.8)	2761 (57.4)
Yes	613 (27.7)	464 (39.6)	326 (37.0)	186 (34.1)	1589 (33.0)
Unsure	249 (11.3)	104 (8.9)	59 (6.7)	50 (9.2)	462 (9.6)
Missing	6	3	2	1	12
Vitamin D test result ^3^					
Normal	372 (60.7)	274 (59.4)	191 (58.6)	109 (58.6)	946 (59.6)
Low	220 (35.9)	172 (37.3)	130 (39.9)	75 (40.3)	597 (37.6)
I wasn’t told/don’t remember	21 (3.4)	15 (3.3)	5 (1.5)	2 (1.1)	43 (2.7)
Missing	0	3	0	0	3

^1^ Home duties, unemployed, student; ^2^ self-reported; ^3^ Includes participants who reported having had a vitamin D test <12 months ago. NT = Northern Territory, QLD = Queensland, ACT = Australia Capital Territory, NSW = New South Wales, VIC = Victoria, TAS = Tasmania, WA = Western Australia, SA = South Australia.

**Table 3:** Associations between personal characteristics and knowledge about/attitudes towards time outdoors needed to avoid vitamin D deficiency.

Personal Characteristics	Time Outdoors in Summer (Over-Estimate vs. Correct) ^1^		Time Outdoors in Winter (Under-Estimate vs. Correct) ^1^		Time Outdoors in Winter (Over-Estimate vs. Correct) ^1^		Sunscreen Stops the Skin from Making Vitamin D (Agree vs. Not Agree)	
	*n* (%) Over-Estimate	APR (95% CI) ^2^	*n* (%) Under-Estimate	APR (95% CI) ^2^	*n* (%) Over-Estimate	APR (95% CI) ^2^	*n* (%) Agree	APR (95% CI) ^2^
State/territory of residence								
QLD/NT	1574 (73.6)	1	566 (26.6)	1	1049 (49.3)	1	425 (19.2)	1
ACT/NSW	872 (76.4)	1.04 (1.00–1.08)	433 (38.1)	0.99 (0.91–1.08)	286 (25.2)	0.60 (0.55–0.66)	296 (25.3)	1.31 (1.15–1.49)
VIC/TAS	715 (82.2)	1.10 (1.06–1.15)	268 (31.0)	0.85 (0.76–0.94)	253 (29.3)	0.64 (0.58–0.70)	220 (25.1)	1.29 (1.12–1.48)
WA/SA	401 (75.4)	1.02 (0.97–1.07)	228 (43.1)	1.09 (0.99–1.21)	126 (23.8)	0.62 (0.54–0.71)	156 (28.6)	1.48 (1.27–1.74)
Age group								
18–39	298 (68.3)	1	176 (40.5)	1	149 (34.3)	1	88 (20.1)	1
40–59	1276 (73.1)	1.06 (0.99–1.14)	625 (35.9)	0.89 (0.80–0.99)	575 (33.0)	0.89 (0.80–1.00)	401 (22.7)	1.15 (0.94–1.41)
60+	1988 (79.5)	1.13 (1.06–1.21)	694 (28.0)	0.79 (0.71–0.88)	990 (39.9)	0.96 (0.86–1.06)	608 (23.4)	1.23 (1.01–1.50)
Sex								
Male	1265 (83.9)	1	342 (22.7)	1	710 (47.2)	1	287 (18.6)	1
Female	2297 (72.3)	0.88 (0.85–0.90)	1153 (36.6)	1.21 (1.10–1.32)	1004 (31.9)	0.84 (0.79–0.89)	810 (24.8)	1.35 (1.20–1.53)
Skin color								
Fair	2427 (74.1)	1	1135 (34.9)	1	1113 (34.2)	1	813 (24.2)	1
Medium	1135 (80.6)	1.07 (1.03–1.10) ^4^	360 (25.6)	0.86 (0.79–0.94)	601 (42.8)	1.07 (1.01–1.14)	284 (19.7)	0.83 (0.74–0.94)
Skin burning								
Burn badly	435 (60.9)	1	337 (47.7)	1	199 (28.1)	1	185 (25.1)	1
Burn moderately	1501 (75.0)	1.22 (1.15–1.30) ^4^	682 (34.3)	0.79 (0.73–0.86)	659 (33.1)	0.95 (0.86–1.05)	508 (24.8)	1.00 (0.87–1.16)
Burn a little	1377 (81.8)	1.31 (1.23–1.39) ^4^	428 (25.5)	0.69 (0.63–0.76)	703 (41.9)	1.05 (0.96–1.16)	330 (19.2)	0.80 (0.68–0.94)
Does not burn	249 (87.7)	1.39 (1.30–1.50) ^4^	47 (16.6)	0.57 (0.45–0.71)	153 (54.1)	1.14 (1.01–1.28)	74 (25.4)	1.05 (0.83–1.33)
Missing	1		1		0		1	
Educational attainment								
No post-school qualification	638 (83.4)	1	178 (23.4)	1	374 (49.1)	1	131 (16.4)	1
Trade/apprenticeship/diploma	862 (79.1)	0.94 (0.90–0.98) ^4^	288 (26.6)	1.06 (0.92–1.21)	467 (43.2)	0.94 (0.88–1.02)	222 (19.9)	1.22 (1.01–1.49)
University degree	2062 (72.9)	0.87 (0.84–0.91) ^4^	1029 (36.6)	1.15 (1.03–1.30)	873 (31.0)	0.82 (0.77–0.88)	744 (25.8)	1.55 (1.31–1.83)
Ever had melanoma removed ^3^								
No	2987 (76.3)	1	1258 (32.3)	1	1395 (35.8)	1	936 (23.4)	1
Yes	508 (73.6)	0.94 (0.90–0.99) ^4^	221 (32.3)	1.13 (1.02–1.24)	277 (40.5)	1.01 (0.93–1.09)	148 (20.6)	0.93 (0.79–1.08)
Missing	79		38		64		88	
Number of skin cancers excised ^3^								
None	1996 (75.2)	1	887 (33.6)	1	899 (34.0)	1	653 (24.2)	1
1	464 (76.4)	1.01 (0.96–1.06) ^4^	186 (30.8)	1.01 (0.91–1.13)	227 (37.6)	1.00 (0.91–1.10)	161 (25.8)	1.03 (0.89–1.20)
2+	1097 (77.5)	1.00 (0.96–1.03) ^4^	421 (30.0)	1.08 (0.99–1.18)	587 (41.8)	1.05 (0.98–1.13)	279 (18.9)	0.80 (0.70–0.91)
Missing	6		5		5		6	

^1^ Number of minutes needed to stay outdoors between 9am and 3pm each day to avoid having low vitamin D; ^2^ Unless otherwise specified, adjusted prevalence ratios (APRs) (95% confidence interval (CIs)) were estimated using log-binomial model. Models with state/territory of residence, age and sex are mutually adjusted for each other. Models with skin color, skin type are adjusted for state/territory of residence, age, sex and educational attainment. Educational attainment is adjusted for state/territory of residence, age, sex and skin color. History of melanoma and skin cancer treatment are adjusted for state/territory, age, sex, skin color and educational attainment; ^3^ self-reported; ^4^ Estimates are from Poisson regression since the log-binomial model was unable to estimate the asymptotic covariance matrix. NT = Northern Territory, QLD = Queensland, ACT = Australia Capital Territory, NSW = New South Wales, VIC = Victoria, TAS = Tasmania, WA = Western Australia, SA = South Australia.

**Table 4:** Associations between knowledge about/attitudes towards vitamin D/sun exposure and sun protection behaviors.

Knowledge about/Attitudes towards Vitamin D and Sun Exposure	Wear Hat if Outdoors for ≥30 min in Summer ^1^		Apply Sunscreen if Outdoors for ≥30 min in Summer ^1^		Routinely Apply Sunscreen to Face ^2^		Routinely Apply Sunscreen to Arms/Other Parts ^2^	
	*n* (%)	APR (95% CI) ^3^	*n* (%)	APR (95% CI) ^3^	*n* (%)	APR (95% CI) ^3^	*n* (%)	APR (95% CI) ^3^
Time outdoors needed in summer ^4^								
Correct	814 (73.6)	1	755 (68.2)	1	676 (61.5)	1	296 (41.2)	1
Over-estimate	2268 (64.5)	0.86 (0.82–0.89) ^5^	1794 (51.1)	0.85 (0.81–0.89)	1867 (53.8)	0.99 (0.94–1.04)	823 (33.8)	0.92 (0.84–1.02)
Missing	136		139		139		95	
Time outdoors needed in winter ^4^								
Correct	925 (64.6)	1	815 (57.0)	1	795 (56.4)	1	353 (36.4)	1
Under-estimate	1059 (71.8)	1.13 (1.08–1.19) ^5^	987 (66.9)	1.09 (1.03–1.15)	896 (61.0)	1.01 (0.95–1.06)	386 (40.2)	1.04 (0.93–1.15)
Over-estimate	1084 (64.1)	0.95 (0.90–1.00) ^5^	732 (43.3)	0.81 (0.76–0.87)	836 (50.2)	0.98 (0.93–1.04)	372 (30.9)	0.97 (0.86–1.08)
Missing	162		166		165		113	
Sunscreen stops the skin from making vitamin D								
Not agree	2429 (66.5)	1	1966 (53.8)	1	2003 (55.4)	1	884 (35.3)	1
Agree	734 (67.7)	1.00 (0.96–1.04)	637 (58.8)	1.05 (0.99–1.10)	608 (56.9)	0.96 (0.91–1.01)	261 (36.0)	0.92 (0.83–1.02)
Missing	22		22		22		20	
Taking vitamin D supplement								
No	2236 (68.2)	1	1782 (54.3)	1	1715 (52.8)	1	788 (33.9)	1
Yes	881 (63.3)	0.96 (0.92–1.00)	796 (57.2)	1.01 (0.96–1.06)	863 (62.7)	1.05 (1.00–1.10)	352 (40.5)	0.99 (0.90–1.09)
Missing	87		86		86		56	
Had vitamin D test in the past 12 months								
No	1884 (69.1)	1	1502 (55.1)	1	1452 (53.9)	1	650 (34.3)	1
Yes	974 (62.1)	0.95 (0.91–0.99)	892 (56.9)	0.99 (0.94–1.04)	958 (61.6)	1.00 (0.96–1.05)	403 (40.1)	0.98 (0.89–1.08)
Unsure	311 (68.8)	0.98 (0.93–1.04)	218 (47.9)	0.92 (0.84–1.01)	210 (46.8)	0.90 (0.82–0.99)	101 (29.7)	0.87 (0.74–1.03)
Missing	12		12		11		9	

^1^ Almost all/all of the time versus never/rarely/sometimes; ^2^ Yes versus no; ^3^ Unless otherwise specified, adjusted prevalence ratios (APRs) (95% confidence interval (CIs)) were estimated using log-binomial model. All are adjusted for state/territory of residence, age, sex, skin color and educational attainment; ^4^ Number of minutes needed to stay outdoors between 9am and 3pm each day to avoid having low vitamin D; ^5^ Estimates are from Poisson regression since the log-binomial model was unable to estimate the asymptotic covariance matrix.

**Table 5:** Associations between knowledge about/attitudes towards vitamin D/sun exposure and changing behaviors to get enough vitamin D.

Knowledge about/Attitudes towards Vitamin D and Sun Exposure	Changed Behavior to Get Enough Vitamin D ^1^		Spend More Time Outdoors ^1,2^		Apply Sunscreen Less ^1,2^		Sunbathe More Often ^1,2^	
	*n* (%) Yes	APR (95% CI) ^3^	*n* (%) yes	APR (95% CI) ^3^	*n* (%) Yes	APR (95% CI) ^3^	*N* (%) Yes	APR (95% CI) ^3^
Time outdoors needed in summer ^4^								
Correct	160 (14.3)	1	107 (66.9)	1	25 (15.6)	1	5 (3.1)	1
Over-estimate	502 (14.1)	1.02 (0.86–1.20)	381 (75.9)	1.16 (1.03–1.31) ^5^	85 (16.9)	1.06 (0.70–1.61)	34 (6.8)	2.01 (0.79–5.13)
Missing	139		24		24		24	
Time outdoors needed in winter ^4^								
Correct	237 (16.4)	1	179 (75.5)	1	38 (16.0)	1	14 (5.9)	1
Under-estimate	231 (15.5)	0.93 (0.78–1.09)	165 (71.4)	0.92 (0.83–1.03) ^5^	34 (14.7)	0.90 (0.59–1.39)	9 (3.9)	0.66 (0.29–1.51)
Over-estimate	189 (11.0)	0.77 (0.64–0.93)	142 (75.1)	1.01 (0.90–1.12) ^5^	37 (19.6)	1.25 (0.83–1.89)	16 (8.5)	1.38 (0.69–2.77)
Missing	166		29		29		29	
Sunscreen stops the skin from making vitamin D								
Not agree	436 (11.8)	1	327 (75.0)	1	40 (9.2)	1	22 (5.0)	1
Agree	247 (22.5)	1.77 (1.54–2.04)	169 (68.4)	0.91 (0.83–1.01) ^5^	74 (30.0)	3.20 (2.25–4.55)	19 (7.7)	1.66 (0.93–2.97)
Missing	22		3		3		3	
Taking vitamin D supplement								
No	394 (11.8)	1	269 (68.3)	1	76 (19.3)	1	27 (6.9)	1
Yes	276 (19.6)	1.49 (1.29–1.72)	215 (77.9)	1.14 (1.04–1.25) ^5^	35 (12.7)	0.63 (0.43–0.92)	14 (5.1)	0.93 (0.50–1.74)
Missing	87		16		16		16	
Had vitamin D test in the past 12 months								
No	296 (10.7)	1	190 (64.2)	1	60 (20.3)	1	17 (5.7)	1
Yes	348 (21.9)	1.85 (1.60–2.14)	280 (80.5)	1.29 (1.16–1.42) ^5^	46 (13.2)	0.60 (0.42–0.86)	23 (6.6)	1.47 (0.80–2.71)
Unsure	42 (9.1)	0.87 (0.64–1.18)	27 (64.3)	1.02 (0.80–1.29) ^5^	8 (19.0)	0.79 (0.40–1.55)	1 (2.4)	0.40 (0.05–2.96)
Missing	11		0		0		0	

^1^ Yes versus no; ^2^ Analysis restricted to those who reported changing their behaviors to get enough vitamin D; ^3^ Unless specified otherwise, adjusted prevalence ratio (APRs) (95% confidence interval (CIs)) were estimated using log-binomial model. All are adjusted for state/territory of residence, age, sex, skin color and educational attainment; ^4^ Number of minutes needed to stay outdoors between 9am and 3pm each day to avoid having low vitamin D; ^5^ Estimates are from Poisson regression since the log-binomial model was unable to estimate the asymptotic covariance matrix.

**Table 6:** Associations between knowledge about/attitudes towards vitamin D/sun exposure and being sunburnt in the past 6 months.

Knowledge about/Attitudes towards Vitamin D and Sun Exposure	Sunburn in the Past 6 Months (Yes vs. No)		
	*n* (%) Yes	CPR (95% CI) ^1^	APR (95% CI) ^2^
Time outdoors needed in summer ^3^			
Correct	359 (32.1)	1	1
Over-estimate	1387 (38.9)	1.21 (1.11–1.34)	1.27 (1.16–1.39) ^4^
Missing	141		
Time outdoors needed in winter ^3^			
Correct	511 (35.3)	1	1
Under-estimate	477 (31.9)	0.90 (0.82–1.00)	0.85 (0.77–0.93) ^4^
Over-estimate	754 (44.0)	1.25 (1.14–1.36)	1.22 (1.12–1.33) ^4^
Missing	168		
Sunscreen stops the skin from making vitamin D			
Not agree	1410 (38.1)	1	1
Agree	373 (34.0)	0.89 (0.81–0.98)	0.94 (0.87–1.02)
Missing	23		
Had vitamin D test in the past 12 months			
No	1098 (39.8)	1	1
Yes	488 (30.7)	0.77 (0.71–0.84)	0.87 (0.80–0.95)
Unsure	200 (43.3)	1.09 (0.97–1.22)	1.11 (1.00–1.23)
Missing	12		
Taking vitamin D supplement			
No	1361 (40.9)	1	1
Yes	398 (28.2)	0.69 (0.63–0.76)	0.81 (0.74–0.88)
Missing	88		

^1^ Crude prevalence ratio (95% confidence interval (CIs)). Models include participants in the adjusted model only; ^2^ Unless otherwise specified, adjusted prevalence ratios (APRs) (95% CIs) were estimated using log-binomial model. All are adjusted for state/territory of residence, age, sex, skin color and educational attainment; ^3^ Number of minutes needed to stay outdoors between 9am and 3pm each day to avoid having low vitamin D; ^4^ Estimates are from Poisson regression since the log-binomial model was unable to estimate the asymptotic covariance matrix.

## Data Availability

The data presented in this study are available in this article (and Supplementary Material).

## Supplementary Materials

The following supporting information can be downloaded at: https://www.mdpi.com/article/10.3390/curroncol30020188/s1, Table S1: Description of personal characteristics, knowledge about/attitude towards vitamin D and sun exposure, sun-related behaviors, by self-reported skin color; Table S2: Description of participants’ knowledge about/attitudes towards vitamin D and sun exposure, sun-related behaviors and sunburn, by state/territory; Table S3: Description of participants’ sun protective behaviors, by personal characteristics; Table S4: Description of participants’ behavior change and sunburn in the past 6 months, by personal characteristics.
