# Supplementary material for: Vitamin D and Sun Exposure: A Community Survey in Australia
Source: Curr Oncol. 2023 Feb 18;30(2):2465–81. doi: 10.3390/curroncol30020188 (PMC9955356; doi:10.3390/curroncol30020188)
Supplement: Supplementary file 1 [file curroncol-30-00188-s001.zip › curroncol-2128489-supplementary.pdf]

SUPPLEMENTARY TABLES

Table S1. Description of personal characteristics, knowledge about/attitude towards vitamin D and sun exposure, sun-related behaviors, by self-reported skin color

Personal characteristics	N (%) Fair (N=3513)	Medium (N=1510)	Olive/dark (N=348)	Total (N=5371)
Age group				
18-39	343 (9.8)	107 (7.2)	40 (11.6)	490 (9.2)
40-59	1312 (37.7)	508 (34.0)	117 (34.0)	1937 (36.4)
60+	1828 (52.5)	877 (58.8)	187 (54.4)	2892 (54.4)
Missing	30	18	4	52
Sex				
Male	1019 (29.5)	558 (37.6)	129 (38.1)	1706 (32.4)
Female	2430 (70.5)	927 (62.4)	210 (61.9)	3567 (67.6)
Missing	64	25	9	98
Educational attainment				
No post-school qualification	566 (16.4)	261 (17.6)	65 (19.2)	892 (16.9)
Trade/apprenticeship/diploma	777 (22.5)	369 (24.9)	89 (26.3)	1235 (23.4)
University degree	2113 (61.1)	854 (57.5)	185 (54.6)	3152 (59.7)
Missing	57	26	9	92
Occupation status				
Full-time worker	1123 (32.5)	416 (28.0)	118 (34.6)	1657 (31.4)
Part-time worker	623 (18.0)	288 (19.4)	50 (14.7)	961 (18.2)
Retired	1380 (40.0)	670 (45.1)	145 (42.5)	2195 (41.6)
Other [1]	327 (9.5)	113 (7.6)	28 (8.2)	468 (8.9)
Missing	60	23	7	90
Ever had melanoma removed [2]				
No	2858 (83.3)	1294 (88.1)	321 (94.4)	4473 (85.3)
Yes	574 (16.7)	175 (11.9)	19 (5.6)	768 (14.7)
Missing	81	41	8	130
Number of skin cancers excised [2]				
None	1808 (51.8)	1010 (67.2)	277 (80.5)	3095 (58.0)
1	441 (12.6)	205 (13.6)	31 (9.0)	677 (12.7)
2+	1238 (35.5)	287 (19.1)	36 (10.5)	1561 (29.3)
Missing	26	8	4	38
Number of skin cancers frozen/burnt [2]				
None	1449 (41.6)	816 (54.4)	241 (70.1)	2506 (47.0)
1-5	923 (26.5)	422 (28.1)	78 (22.7)	1423 (26.7)
6+	1115 (32.0)	263 (17.5)	25 (7.3)	1403 (26.3)
Missing	26	9	4	39
Taking vitamin D supplement				
No	2387 (69.2)	1077 (72.4)	244 (71.8)	3708 (70.3)
Yes	1060 (30.8)	410 (27.6)	96 (28.2)	1566 (29.7)
Missing	66	23	8	97
Had vitamin D tested <12 months ago				
No	2009 (57.4)	879 (58.3)	186 (53.6)	3074 (57.4)
Yes	1157 (33.0)	490 (32.5)	134 (38.6)	1781 (33.2)
Unsure	337 (9.6)	139 (9.2)	27 (7.8)	503 (9.4)
Missing	10	2	1	13
Time outdoors needed in summer [3]				
Correct	874 (25.6)	287 (19.6)	48 (14.4)	1209 (23.2)
Over-estimate	2535 (74.4)	1181 (80.4)	286 (85.6)	4002 (76.8)
Missing	104	42	14	160
Time outdoors needed in winter [3]				
Correct	1027 (30.9)	450 (31.3)	84 (25.8)	1561 (30.7)
Under-estimate	1158 (34.9)	371 (25.8)	70 (21.5)	1599 (31.4)
Over-estimate	1136 (34.2)	618 (42.9)	172 (52.8)	1926 (37.9)
Missing	192	71	22	285
Sunscreen stops the skin from making vitamin D				
Not agree	2659 (76.0)	1203 (80.1)	275 (79.3)	4137 (77.4)
Agree	839 (24.0)	298 (19.9)	72 (20.7)	1209 (22.6)
Missing	15	9	1	25

Personal characteristics	N (%) Fair (N=3513)	Medium (N=1510)	Olive/dark (N=348)	Total (N=5371)
Wear hat if being outdoors for 30 minutes or more in summer				
Never/rarely/sometimes	1036 (30.2)	603 (40.4)	159 (46.6)	1798 (34.2)
Almost all/all of the time	2396 (69.8)	889 (59.6)	182 (53.4)	3467 (65.8)
Missing	81	18	7	106
Apply sunscreen if being outdoors for 30 minutes or more in summer				
Never/rarely/sometimes	1403 (40.7)	823 (55.4)	228 (67.1)	2454 (46.5)
Almost all/all of the time	2046 (59.3)	663 (44.6)	112 (32.9)	2821 (53.5)
Missing	64	24	8	96
Routinely apply sunscreen to face				
No	1399 (41.0)	757 (51.7)	199 (58.7)	2355 (45.1)
Yes	2017 (59.0)	707 (48.3)	140 (41.3)	2864 (54.9)
Missing	97	46	9	152
Routinely apply sunscreen to arms/other parts				
No	1404 (60.6)	759 (72.7)	201 (77.9)	2364 (65.3)
Yes	912 (39.4)	285 (27.3)	57 (22.1)	1254 (34.7)
Missing	1197	466	90	1753
Changed behavior to get enough vitamin D				
No	2997 (85.4)	1304 (86.4)	297 (85.6)	4598 (85.7)
Yes	514 (14.6)	205 (13.6)	50 (14.4)	769 (14.3)
Missing	2	1	1	4
Spend more time outdoors [4]				
No	144 (28.0)	60 (29.3)	10 (20.0)	214 (27.8)
Yes	370 (72.0)	145 (70.7)	40 (80.0)	555 (72.2)
Missing	2999	1305	298	4602
Apply sunscreen less [4]				
No	436 (84.8)	166 (81.0)	42 (84.0)	644 (83.7)
Yes	78 (15.2)	39 (19.0)	8 (16.0)	125 (16.3)
Missing	2999	1305	298	4602
Sunbathe more often [4]				
No	490 (95.3)	186 (90.7)	46 (92.0)	722 (93.9)
Yes	24 (4.7)	19 (9.3)	4 (8.0)	47 (6.1)
Missing	2999	1305	298	4602

[1] Home duties, unemployed, student; [2] self-reported; [3] Number of minutes needed to stay outdoors between 9am and 3pm each day to avoid having low vitamin D; [4] restricted to those who reported changing their behaviors to get enough vitamin D

Table S2. Description of participants' knowledge about/attitudes towards vitamin D and sun exposure, sun-related behaviors, by state/territory

Knowledge about/attitudes towards vitamin D/sun exposure, sun-related behaviors	n (column %) NT/QLD (N=2219)	ACT/NSW (N=1174)	VIC/TAS (N=884)	WA/SA (N=547)	Total (N=4824)
Sources of vitamin D [1]					
Oily fish	940 (42.4)	495 (42.2)	414 (46.8)	241 (44.1)	2090 (43.3)
Exposing the skin to the sun	2162 (97.4)	1143 (97.4)	870 (98.4)	542 (99.1)	4717 (97.8)
Taking vitamin D tablets	1604 (72.3)	885 (75.4)	703 (79.5)	417 (76.2)	3609 (74.8)
Time outdoors needed in summer [2]					
Over-estimate	1574 (73.6)	872 (76.4)	715 (82.2)	401 (75.4)	3562 (76.1)
Correct	566 (26.4)	269 (23.6)	155 (17.8)	131 (24.6)	1121 (23.9)
Missing	79	33	14	15	141
Time outdoors needed in winter [2]					
Under-estimate	566 (26.6)	433 (38.1)	268 (31.0)	228 (43.1)	1495 (32.1)
Over-estimate	1049 (49.3)	286 (25.2)	253 (29.3)	126 (23.8)	1714 (36.8)
Correct	511 (24.0)	418 (36.8)	343 (39.7)	175 (33.1)	1447 (31.1)
Missing	93	37	20	18	168
My vitamin D levels might be low					
Not agree	1912 (86.5)	966 (82.5)	701 (79.4)	444 (81.3)	4023 (83.6)
Agree	298 (13.5)	205 (17.5)	182 (20.6)	102 (18.7)	787 (16.4)
Missing	9	3	1	1	14
Sunscreen stops the skin from making vitamin D					
Not agree	1783 (80.8)	874 (74.7)	657 (74.9)	390 (71.4)	3704 (77.2)
Agree	425 (19.2)	296 (25.3)	220 (25.1)	156 (28.6)	1097 (22.8)
Missing	11	4	7	1	23

Knowledge about/attitudes towards vitamin D/sun exposure, sun-related behaviors	n (column %)				
	NT/QLD (N=2219)	ACT/NSW (N=1174)	VIC/TAS (N=884)	WA/SA (N=547)	Total (N=4824)
Going outside early in the morning makes enough vitamin D					
Not agree	1218 (55.3)	763 (65.3)	670 (76.0)	350 (64.2)	3001 (62.5)
Agree	986 (44.7)	405 (34.7)	211 (24.0)	195 (35.8)	1797 (37.5)
Missing	15	6	3	2	26
It's OK to risk getting skin cancer to avoid low vitamin D					
Not agree	2155 (98.0)	1132 (96.9)	861 (97.8)	534 (98.2)	4682 (97.7)
Agree	45 (2.0)	36 (3.1)	19 (2.2)	10 (1.8)	110 (2.3)
Missing	19	6	4	3	32
Changed behavior to get enough vitamin D					
No	1975 (89.0)	980 (83.6)	746 (84.4)	434 (79.3)	4135 (85.8)
Yes	243 (11.0)	192 (16.4)	138 (15.6)	113 (20.7)	686 (14.2)
Missing	1	2	0	0	3
Apply sunscreen if being outdoors 30 minutes or more in summer					
Never/rarely/sometimes	1008 (46.0)	513 (44.2)	382 (43.9)	239 (44.3)	2142 (45.0)
Almost all/all of the time	1181 (54.0)	648 (55.8)	488 (56.1)	301 (55.7)	2618 (55.0)
Missing	30	13	14	7	64
Wear hat if being outdoors 30 minutes or more in summer					
Never/rarely/sometimes	626 (28.6)	414 (35.8)	351 (40.1)	188 (34.8)	1579 (33.2)
Almost all/all of the time	1559 (71.4)	743 (64.2)	524 (59.9)	352 (65.2)	3178 (66.8)
Missing	34	17	9	7	67
Wear long-sleeves if being outdoors 30 minutes or more in summer					
Never/rarely/sometimes	1728 (80.2)	963 (83.6)	671 (77.7)	429 (80.2)	3791 (80.6)
Almost all/all of the time	426 (19.8)	189 (16.4)	193 (22.3)	106 (19.8)	914 (19.4)
Missing	65	22	20	12	119
Routinely apply sunscreen to face					
No	1007 (46.5)	467 (40.7)	370 (42.9)	239 (44.9)	2083 (44.2)
Yes	1159 (53.5)	681 (59.3)	493 (57.1)	293 (55.1)	2626 (55.8)
Missing	53	26	21	15	115
Routinely apply sunscreen to arms/other parts					
No	1011 (67.5)	467 (61.7)	369 (60.3)	242 (63.5)	2089 (64.3)
Yes	486 (32.5)	290 (38.3)	243 (39.7)	139 (36.5)	1158 (35.7)
Missing	722	417	272	166	1577

[1] Multiple choice question; [2] Number of minutes needed to stay outdoors between 9am and 3pm each day to avoid having low vitamin D; NT = Northern Territory, QLD = Queensland, ACT = Australia Capital Territory, NSW = New South Wales, VIC = Victoria, TAS = Tasmania, WA = Western Australia, SA = South Australia.

Table S3. Description of participants' sun protective behaviors, by personal characteristics

Personal characteristics	n (row %)							
	Wear hat if being outdoors for 30 minutes in summer		Apply sunscreen if being outdoors for 30 minutes in summer		Routinely apply sunscreen to face		Routinely apply sunscreen to arms/other parts	
	N/R/S [1] (N=1579)	AOTT [2] (N=3178)	N/R/S [1] (N=2142)	AOTT [2] (N=2618)	No (N=2083)	Yes (N=2626)	No (N=2089)	Yes (N=1158)
State/territory of residence								
NT/QLD	626 (28.6)	1559 (71.4)	1008 (46.0)	1181 (54.0)	1007 (46.5)	1159 (53.5)	1011 (67.5)	486 (32.5)
NSW/ACT	414 (35.8)	743 (64.2)	513 (44.2)	648 (55.8)	467 (40.7)	681 (59.3)	467 (61.7)	290 (38.3)
VIC/TAS	351 (40.1)	524 (59.9)	382 (43.9)	488 (56.1)	370 (42.9)	493 (57.1)	369 (60.3)	243 (39.7)
WA/SA	188 (34.8)	352 (65.2)	239 (44.3)	301 (55.7)	239 (44.9)	293 (55.1)	242 (63.5)	139 (36.5)
Sex								
Male	316 (20.7)	1212 (79.3)	895 (59.1)	619 (40.9)	1095 (73.0)	406 (27.0)	1093 (78.2)	305 (21.8)
Female	1263 (39.1)	1966 (60.9)	1247 (38.4)	1999 (61.6)	988 (30.8)	2220 (69.2)	996 (53.9)	853 (46.1)
Age group								
18-39	231 (52.7)	207 (47.3)	146 (33.3)	292 (66.7)	184 (42.6)	248 (57.4)	185 (73.1)	68 (26.9)
40-59	614 (35.1)	1137 (64.9)	691 (39.2)	1070 (60.8)	691 (39.8)	1046 (60.2)	694 (63.5)	399 (36.5)
60+	734 (28.6)	1834 (71.4)	1305 (51.0)	1256 (49.0)	1208 (47.6)	1332 (52.4)	1210 (63.7)	691 (36.3)
Educational attainment								
No post-school qualification	315 (40.0)	472 (60.0)	429 (54.2)	363 (45.8)	367 (46.9)	415 (53.1)	370 (68.5)	170 (31.5)
Trade/apprenticeship/diploma	372 (33.7)	733 (66.3)	585 (53.1)	517 (46.9)	513 (46.6)	587 (53.4)	513 (65.4)	271 (34.6)
University degree	892 (31.1)	1973 (68.9)	1128 (39.4)	1738 (60.6)	1203 (42.6)	1624 (57.4)	1206 (62.7)	717 (37.3)
Occupation status								
Full-time worker	544 (36.6)	942 (63.4)	609 (40.8)	883 (59.2)	648 (44.1)	821 (55.9)	651 (68.0)	306 (32.0)
Part-time worker	306 (35.0)	569 (65.0)	336 (38.4)	540 (61.6)	293 (33.6)	579 (66.4)	292 (58.6)	206 (41.4)
Retired	544 (27.6)	1428 (72.4)	1015 (51.6)	952 (48.4)	950 (48.7)	1000 (51.3)	954 (63.6)	546 (36.4)
Other [3]	185 (44.4)	232 (55.6)	179 (42.8)	239 (57.2)	190 (46.2)	221 (53.8)	190 (66.2)	97 (33.8)
Missing	0	7	3	4	2	5	2	3
Skin color								
Fair	1001 (30.1)	2320 (69.9)	1353 (40.6)	1977 (59.4)	1351 (40.9)	1949 (59.1)	1355 (60.5)	883 (39.5)
Medium	578 (40.3)	858 (59.7)	789 (55.2)	641 (44.8)	732 (52.0)	677 (48.0)	734 (72.7)	275 (27.3)
Skin burning								
Burn badly	156 (21.4)	573 (78.6)	185 (25.5)	540 (74.5)	234 (32.6)	483 (67.4)	235 (47.8)	257 (52.2)
Burn moderately	609 (30.0)	1423 (70.0)	787 (38.7)	1248 (61.3)	822 (40.9)	1190 (59.1)	825 (61.7)	512 (38.3)
Burn a little	667 (39.1)	1040 (60.9)	964 (56.3)	748 (43.7)	845 (50.0)	846 (50.0)	846 (71.0)	346 (29.0)
Does not burn	147 (51.0)	141 (49.0)	206 (71.8)	81 (28.2)	182 (63.2)	106 (36.8)	183 (81.0)	43 (19.0)
Missing	0	1	0	1	0	1	0	0
Ever had melanoma removed								
No	1369 (34.6)	2591 (65.4)	1788 (45.1)	2177 (54.9)	1731 (44.1)	2193 (55.9)	1737 (65.2)	928 (34.8)
Yes	170 (24.0)	538 (76.0)	308 (43.6)	399 (56.4)	307 (44.0)	391 (56.0)	307 (60.1)	204 (39.9)
Missing	40	49	46	42	45	42	45	26

Personal characteristics	n (row %)							
	Wear hat if being outdoors for 30 minutes in summer		Apply sunscreen if being outdoors for 30 minutes in summer		Routinely apply sunscreen to face		Routinely apply sunscreen to arms/other parts	
	N/R/S [1] (N=1579)	AOTT [2] (N=3178)	N/R/S [1] (N=2142)	AOTT [2] (N=2618)	No (N=2083)	Yes (N=2626)	No (N=2089)	Yes (N=1158)
Number of skin cancers ever excised								
None	1069 (39.8)	1614 (60.2)	1230 (45.8)	1457 (54.2)	1223 (46.3)	1421 (53.7)	1226 (68.4)	566 (31.6)
1	198 (32.2)	417 (67.8)	297 (47.8)	324 (52.2)	256 (41.6)	360 (58.4)	258 (62.3)	156 (37.7)
2+	311 (21.4)	1142 (78.6)	615 (42.5)	831 (57.5)	601 (41.6)	842 (58.4)	602 (58.1)	434 (41.9)
Missing	1	5	0	6	3	3	3	2
Number of skin cancers ever frozen/burnt								
None	938 (43.5)	1216 (56.5)	993 (46.0)	1168 (54.0)	964 (45.2)	1167 (54.8)	967 (68.2)	451 (31.8)
1-5	364 (28.6)	910 (71.4)	603 (47.3)	673 (52.7)	554 (43.9)	708 (56.1)	556 (64.4)	308 (35.6)
6+	274 (20.7)	1048 (79.3)	542 (41.2)	774 (58.8)	563 (43.0)	746 (57.0)	564 (58.6)	398 (41.4)
Missing	3	4	4	3	2	5	2	1

[1] Never/rarely/sometimes; [2] Almost all/all of the time; [3] Home duties, unemployed, student; NT = Northern Territory, QLD = Queensland, ACT = Australia Capital Territory, NSW = New South Wales, VIC = Victoria, TAS = Tasmania, WA = Western Australia, SA = South Australia.

Table S4. Description of participants' behavior change and sunburn in the past 6 months, by personal characteristics

Personal characteristics	n (row %)									
	Changed behavior to get enough vitamin D		Spend more time outdoors [1]		Apply sunscreen less [1]		Sunbathe more often [1]		Sunburn in the last 6 months [1]	
	No (N=4135)	Yes (N=686)	No (N=189)	Yes (N=497)	No (N=572)	Yes (N=114)	No (N=645)	Yes (N=41)	No (N=3035)	Yes (N=1788)
State/territory of residence										
NT/QLD	1975 (89.0)	243 (11.0)	72 (29.6)	171 (70.4)	207 (85.2)	36 (14.8)	229 (94.2)	14 (5.8)	1368 (61.7)	850 (38.3)
NSW/ACT	980 (83.6)	192 (16.4)	51 (26.6)	141 (73.4)	159 (82.8)	33 (17.2)	180 (93.8)	12 (6.2)	768 (65.4)	406 (34.6)
VIC/TAS	746 (84.4)	138 (15.6)	40 (29.0)	98 (71.0)	110 (79.7)	28 (20.3)	128 (92.8)	10 (7.2)	567 (64.1)	317 (35.9)
WA/SA	434 (79.3)	113 (20.7)	26 (23.0)	87 (77.0)	96 (85.0)	17 (15.0)	108 (95.6)	5 (4.4)	332 (60.7)	215 (39.3)
Sex										
Male	1387 (89.8)	158 (10.2)	48 (30.4)	110 (69.6)	130 (82.3)	28 (17.7)	140 (88.6)	18 (11.4)	891 (57.7)	654 (42.3)
Female	2748 (83.9)	528 (16.1)	141 (26.7)	387 (73.3)	442 (83.7)	86 (16.3)	505 (95.6)	23 (4.4)	2144 (65.4)	1134 (34.6)
Age group										
18-39	387 (88.4)	51 (11.6)	4 (7.8)	47 (92.2)	47 (92.2)	4 (7.8)	46 (90.2)	5 (9.8)	138 (31.5)	300 (68.5)
40-59	1524 (86.0)	248 (14.0)	69 (27.8)	179 (72.2)	212 (85.5)	36 (14.5)	234 (94.4)	14 (5.6)	942 (53.1)	831 (46.9)
60+	2224 (85.2)	387 (14.8)	116 (30.0)	271 (70.0)	313 (80.9)	74 (19.1)	365 (94.3)	22 (5.7)	1955 (74.8)	657 (25.2)
Educational attainment										
No post-school qualification	695 (86.7)	107 (13.3)	30 (28.0)	77 (72.0)	95 (88.8)	12 (11.2)	103 (96.3)	4 (3.7)	505 (62.9)	298 (37.1)
Trade/apprenticeship/ diploma	965 (85.7)	161 (14.3)	42 (26.1)	119 (73.9)	130 (80.7)	31 (19.3)	151 (93.8)	10 (6.2)	695 (61.8)	430 (38.2)
University degree	2475 (85.6)	418 (14.4)	117 (28.0)	301 (72.0)	347 (83.0)	71 (17.0)	391 (93.5)	27 (6.5)	1835 (63.4)	1060 (36.6)

Personal characteristics	n (row %)									
	Changed behavior to get enough vitamin D		Spend more time outdoors [1]		Apply sunscreen less [1]		Sunbathe more often [1]		Sunburn in the last 6 months [1]	
	No (N=4135)	Yes (N=686)	No (N=189)	Yes (N=497)	No (N=572)	Yes (N=114)	No (N=645)	Yes (N=41)	No (N=3035)	Yes (N=1788)
Occupation status										
Full-time worker	1308 (87.2)	192 (12.8)	55 (28.6)	137 (71.4)	166 (86.5)	26 (13.5)	177 (92.2)	15 (7.8)	746 (49.7)	754 (50.3)
Part-time worker	756 (85.3)	130 (14.7)	34 (26.2)	96 (73.8)	107 (82.3)	23 (17.7)	126 (96.9)	4 (3.1)	533 (60.1)	354 (39.9)
Retired	1714 (85.4)	292 (14.6)	85 (29.1)	207 (70.9)	235 (80.5)	57 (19.5)	277 (94.9)	15 (5.1)	1529 (76.2)	478 (23.8)
Other [2]	350 (82.9)	72 (17.1)	15 (20.8)	57 (79.2)	64 (88.9)	8 (11.1)	65 (90.3)	7 (9.7)	221 (52.4)	201 (47.6)
Missing	7	0	0	0	0	0	0	0	6	1
Skin color										
Fair	2885 (85.5)	489 (14.5)	132 (27.0)	357 (73.0)	414 (84.7)	75 (15.3)	467 (95.5)	22 (4.5)	2078 (61.6)	1297 (38.4)
Medium	1250 (86.4)	197 (13.6)	57 (28.9)	140 (71.1)	158 (80.2)	39 (19.8)	178 (90.4)	19 (9.6)	957 (66.1)	491 (33.9)
Skin burning										
Burn badly	625 (84.2)	117 (15.8)	43 (36.8)	74 (63.2)	97 (82.9)	20 (17.1)	114 (97.4)	3 (2.6)	471 (63.5)	271 (36.5)
Burn moderately	1764 (85.8)	293 (14.2)	80 (27.3)	213 (72.7)	249 (85.0)	44 (15.0)	276 (94.2)	17 (5.8)	1209 (58.7)	850 (41.3)
Burn a little	1491 (86.2)	239 (13.8)	58 (24.3)	181 (75.7)	195 (81.6)	44 (18.4)	221 (92.5)	18 (7.5)	1135 (65.6)	595 (34.4)
Does not burn	254 (87.3)	37 (12.7)	8 (21.6)	29 (78.4)	31 (83.8)	6 (16.2)	34 (91.9)	3 (8.1)	219 (75.3)	72 (24.7)
Missing	1	0	0	0	0	0	0	0	1	0
Ever had melanoma removed [3]										
No	3424 (85.3)	589 (14.7)	155 (26.3)	434 (73.7)	492 (83.5)	97 (16.5)	553 (93.9)	36 (6.1)	2505 (62.4)	1510 (37.6)
Yes	635 (88.3)	84 (11.7)	30 (35.7)	54 (64.3)	68 (81.0)	16 (19.0)	80 (95.2)	4 (4.8)	481 (66.9)	238 (33.1)
Missing	76	13	4	9	12	1	12	1	49	40
Number of skin cancers ever excised [3]										
None	2312 (85.4)	395 (14.6)	92 (23.3)	303 (76.7)	333 (84.3)	62 (15.7)	369 (93.4)	26 (6.6)	1616 (59.7)	1093 (40.3)
1	522 (83.1)	106 (16.9)	32 (30.2)	74 (69.8)	85 (80.2)	21 (19.8)	103 (97.2)	3 (2.8)	431 (68.7)	196 (31.3)
2+	1296 (87.6)	184 (12.4)	65 (35.3)	119 (64.7)	153 (83.2)	31 (16.8)	172 (93.5)	12 (6.5)	983 (66.4)	498 (33.6)
Missing	5	1	0	1	1	0	1	0	5	1
Number of skin cancers ever frozen/burnt [3]										
None	1870 (85.8)	310 (14.2)	75 (24.2)	235 (75.8)	264 (85.2)	46 (14.8)	287 (92.6)	23 (7.4)	1252 (57.5)	927 (42.5)
1-5	1088 (84.1)	206 (15.9)	53 (25.7)	153 (74.3)	172 (83.5)	34 (16.5)	201 (97.6)	5 (2.4)	861 (66.5)	434 (33.5)
6+	1171 (87.4)	169 (12.6)	61 (36.1)	108 (63.9)	135 (79.9)	34 (20.1)	156 (92.3)	13 (7.7)	916 (68.3)	426 (31.7)
Missing	6	1	0	1	1	0	1	0	6	1

[1] Restricted to those who reported changing their behaviors to get enough vitamin D; [2] Home duties, unemployed, student; [3] self-reported; NT = Northern Territory, QLD = Queensland, ACT = Australia Capital Territory, NSW = New South Wales, VIC = Victoria, TAS = Tasmania, WA = Western Australia, SA = South Australia.
